# Adverse events among high-risk participants in a home-based walking study: a descriptive study

**DOI:** 10.1186/1479-5868-4-20

**Published:** 2007-05-23

**Authors:** David E Goodrich, Angela R Larkin, Julie C Lowery, Robert G Holleman, Caroline R Richardson

**Affiliations:** 1 HSR&D Center for Excellence, VA Health Care Medical Center, P.O. Box 130170, Ann Arbor, MI 48113-0170, USA; 2 Department of Family Medicine, University of Michigan, 1018 Fuller St. SPC: 5708, Ann Arbor, MI 48109-5708, USA

## Abstract

**Background:**

For high-risk individuals and their healthcare providers, finding the right balance between promoting physical activity and minimizing the risk of adverse events can be difficult. More information on the prevalence and influence of adverse events is needed to improve providers' ability to prescribe effective and safe exercise programs for their patients.

**Methods:**

This study describes the type and severity of adverse events reported by participants with cardiovascular disease or at-risk for cardiovascular disease that occurred during an unsupervised, home-based walking study. This multi-site, randomized controlled trial tested the feasibility of a diet and lifestyle activity intervention over 1.5 years. At month 13, 274 eligible participants (male veterans) were recruited who were ambulatory, BMI > 28, and reporting one or more cardiovascular disease risk factors. All participants attended five, face-to-face dietitian-delivered counseling sessions during the six-month intervention. Participants were randomized to three study arms: 1) time-based walking goals, 2) simple pedometer-based walking goals, and 3) enhanced pedometer-based walking goals with Internet-mediated feedback. Two physicians verified adverse event symptom coding.

**Results:**

Enrolled participants had an average of five medical comorbidities. During 1110 person months of observation, 87 of 274 participants reported 121 adverse events. One serious study-related adverse event (atrial fibrillation) was reported; the individual resumed study participation within three days. Non-serious, study related adverse events made up 12% of all symptoms – predominantly minor musculoskeletal events. Serious, non-study related adverse events represented 32% of all symptoms while non-serious, non-study related adverse events made up 56% of symptoms. Cardiovascular disease events represented over half of the non-study related adverse event symptoms followed by musculoskeletal complaints. Adverse events caused 50 temporary suspensions averaging 26 days in duration before physician medical clearance was obtained to resume walking.

**Conclusion:**

Men at high risk for adverse cardiovascular events can safely be advised to start a progressive walking program. Results suggest that minor to serious medical problems unrelated to exercise are a major barrier to walking adherence. Helping individuals with chronic illness return to physical activity quickly but safely after an adverse event is an important component of any physical activity intervention targeting this population.

## Background

Exercise, particularly vigorous exercise, increases the risk of adverse cardiovascular events acutely but over the long term, individuals who exercise regularly are at lower risk for these events than those who remain sedentary [1-5]. The risk of an adverse cardiovascular event occurring during or following an acute bout of physical activity (PA) is greatest among new exercisers who are formerly sedentary, middle- to older-aged, and/or who display certain disease risk factors [1,6]. Although there is an elevated risk for serious adverse events (AE's) such as acute myocardial infarctions or sudden cardiac death among sedentary individuals starting a vigorous PA program, this risk is attenuated over time with regular PA participation and increased levels of fitness [1,2,7-10]. Individuals with multiple cardiac risk factors live longer if they are physically active [5]. For high-risk individuals and their healthcare providers, finding the right balance between promoting PA and minimizing the risk of adverse cardiovascular events can be difficult. Exercise in a supervised and monitored setting such as a formal cardiovascular rehabilitation program is often recommended for those at highest risk as in the case of those who have recently had a heart attack or those with congestive heart failure [11-14]. However, many high-risk individuals do not qualify for cardiovascular rehabilitation programs or do not have access to or insurance coverage for such programs [15,16].

Cardiovascular risk is not the only risk associated with starting an exercise program. Minor musculoskeletal injuries including sprains, strains, joint pain, and overuse injuries are common among sedentary people who try to start an exercise program [17]. Musculoskeletal risk is likely to be higher for people who are particularly unfit or obese who are starting a PA program. Additionally, there are medication-related problems for individuals with multiple comorbidities [18]. Individuals with diabetes may experience low blood sugar after an episode of PA, and individuals taking multiple blood pressure medications may need to decrease their blood pressure medications to avoid low blood pressure as they become more active. For many high-risk individuals, concerns or uncertainty about the safety of engaging in PA are valid exercise barriers and may be, in part, attributed to sensational media accounts of cardiac events occurring after vigorous PA participation [1,19]. Similarly, despite the growing evidence of the safety of mild to moderate PA for high-risk populations, some physicians are reluctant to prescribe PA due to the legal concerns of a patient experiencing a serious medical event [15,20]. One approach to reduce the occurrence of minor and severe exercise-related medical events is the prescription of progressive exercise programs emphasizing mild to moderate intensity physical activities like walking that can be easily integrated into a person's lifestyle.

A home-based walking program may be a safe and effective way for high-risk individuals to increase their PA [11,21]. Walking is a moderate-intensity form of PA that carries less risk for adverse cardiovascular or musculoskeletal events than more vigorous forms of PA. However, we know very little about the risks involved with such unsupervised lifestyle activity programs [20,22]. Many PA intervention studies explicitly exclude high-risk individuals. Those studies that included high-risk individuals rarely provide detailed information about adverse events that occurred during the course of the intervention, particularly for community-based studies featuring lifestyle activity interventions [20]. If the public health goal for exercise prescription is to promote long-term PA participation in high-risk groups, healthcare providers not only need an evidence-base that documents the influence of cardiovascular events and musculoskeletal injuries to improve program prescription,[20] but also evidence of the impact of non-intervention-related morbidity and transient illness on exercise adherence.

In this manuscript, we present adverse event (AE) data from a multi-center randomized controlled trial (RCT) of a lifestyle modification program for male veterans with cardiac risk factors. We present a detailed analysis of serious intervention-related adverse events, non-serious intervention-related events, and both serious and non-serious events unrelated to the intervention. This information may be useful to the medical care provider prescribing a home-based walking program for high-risk patients as well as for researchers or program developers who are designing safe, home-based walking programs for these individuals. Additionally, to enhance future PA research that involves unsupervised PA interventions or samples with high levels of disease morbidity, we provide a detailed description of the intervention protocols used to insure participant safety and monitoring of AE's.

## Methods

### Research design

This study is a descriptive analysis of the adverse events in Veterans Walk for Health, a multi-site RCT with three intervention arms. Participants were randomized to one of three study groups: (1) five-session nutritional counseling program with time-based walking goals (i.e., control); (2) five-session nutritional counseling program + simple pedometer feedback; and (3) five-session nutritional counseling program + enhanced pedometer (web-based) feedback. All participants were followed for six months.

### Study population

Individuals with cardiovascular (CVD) risk factors or known CVD were recruited from six Department of Veterans Affairs (VA) medical centers located across the United States. Participants were male VA patients (= age 18) who were either referred by a VA physician for outpatient nutritional counseling or who responded to advertisements for the study posted at each site. Individuals were eligible if they had a body mass index (BMI) = 28, were ambulatory as demonstrated by the ability to comfortably walk for at least one block, and reported at least one major risk factor for CVD including diabetes, coronary artery disease, hypercholesterolemia, hypertension, or obesity. Participants identified a VA physician who could give medical clearance for study participation; no cardiovascular screening tests were conducted as part of the inclusion criteria. Eligible individuals were required to be able to communicate clearly in English and to be sedentary. Sedentary was defined as self-report of performing less than 30 minutes per day of moderate intensity PA on five or more days per week. Eligible participants also needed to express willingness to try a walking program. Finally, participants were excluded if they reported using a pedometer during the 28 days prior to enrollment.

### Intervention

Each participant met with a dietitian over a six-month period for five face-to-face counseling sessions on diet and PA. All participants received behavioral counseling on dietary change using an adaptation of the Medical Nutrition Therapy Protocol for Weight Management (MNT protocol) published by the American Dietetics Association [23]. The intervention protocol for this study was intended to encourage the combination of calorie reduction and walking for weight loss. Participants in the control group received standard dietary counseling and were encouraged to set short, time-based walking goals for exercise. Daily walking time was recorded in a log that was reviewed at each counseling session, and new time-based walking goals were set. Individuals in the simple pedometer group received the MNT protocol and also were taught to self-monitor and track their daily walking levels using a Yamax Digiwalker 200 pedometer. At each counseling session, the dietitian and participant reviewed logged step-count data and revised short- and long-term walking goals with the aim of progressively increasing daily walking. In the enhanced pedometer group, participants' MNT counseling was paired with an "enhanced" SportBrain pedometer. The SportBrain pedometer not only permitted participants to obtain conventional step-count feedback but also allowed the weekly upload of time-stamped pedometer data to a website. The website then generated personalized and graphical analysis (by time of day) of progress towards attainment of daily walking goals.

### Safety protocols

Safety protocols were implemented to minimize the risk for AE's occurring in response to the walking program. Study protocols and forms for the research staff and participants can be found on the study website [24]. First, all participants were required to obtain medical clearance from a VA physician prior to the start of PA counseling (session 2) using a standardized form provided by the research staff after an orientation session explaining the study objectives [see Additional file 1]. Unlike some PA trials that often exclude chronically diseased individuals, Veterans Walk for Health specifically sought out at-risk individuals whose risks were already being managed through regular physician contact. This step assured that the participant was under medical supervision and that his physician viewed walking as a safe and beneficial activity given the participant's medical history and present health status. Additionally, the medical clearance form [see Additional file 1] provided physicians with the option to: 1) endorse study participation; 2) deny participation due to known risks; 3) postpone clearance contingent on further medical assessment; 4) or acknowledge that they lacked adequate knowledge of the patient's medical history to make an informed decision on participation. Participants were not permitted to start the walking intervention until written medical clearance was obtained. Second, each participant was counselled to slowly and realistically increase the speed and duration of their walking program goals to reduce the risk of musculoskeletal injury and adverse CVD events. Dietitians also reviewed a walking handout, which gave instructions on warming up, stretching, and exercise progression. Third, participants with diabetes were provided with a handout on exercising safely with diabetes, developed by the American Academy of Family Physicians and the American Diabetes Association. Finally, if a participant reported either a serious AE or a concerning contraindication to the walking program, the participant was suspended from the study until written medical clearance was provided by the participant's physician again to resume study participation [see Additional file 2]. To minimize study attrition, the study coordinator at the participant's site assisted suspended participants in obtaining medical clearance.

This study was approved by the Investigational Review Boards at each of the six participating VA medical centers in Memphis, Tennessee; Miami, Florida; Oklahoma City, Oklahoma; San Diego, California; Tucson, Arizona; and Topeka, Kansas as well as the coordinating VA medical center in Ann Arbor, Michigan. All eligible participants signed written informed consent documents prior to enrollment.

### Monitoring and classification of adverse events

Due to the high risk for adverse CVD events such as heart attacks and stroke among participants, all members of the research team were charged with the responsibility to protect study participants. Any research staff person who had any reason for concern about the safety of a participant, no matter how small, was required to report the concern immediately to either the site principal investigator or to the study principal investigator. Every dietitian and site coordinator in the study had the authority to suspend participation until medical clearance was re-obtained. Site coordinators and dietitians were responsible for notifying the Ann Arbor coordinator of all AE's using a standardized form and checklist to assure compliance with Internal Review Board (IRB) safety protocols [see Additional file 3]. AE reports were transmitted to the coordinating site by fax or PDF electronic file, insuring that all participant data was kept confidential by assigning numerical codes to participant and site. All AE reports were reviewed by the study's principal investigator; a physician who confirmed AE coding. The Ann Arbor study coordinator maintained a master log of all study-related AE's. The AE report form also provided research coordinators at each site with a means to track temporary study suspensions due to AE's and update the coordinating site regarding the resumption of study participation for suspended individuals.

### Coding of adverse events

To insure consistent AE coding, all AE report forms were analyzed and coded by two physicians at the central site according to the type(s) and severity of adverse medical symptoms. Coding discrepancies were discussed and resolved. Symptoms were classified as either possibly study-related or probably not study-related. Adverse cardiovascular events or symptoms that occurred during or immediately following a walk or in the six hours following an episode of walking were considered study related. In some cases, investigators used professional judgement to code study-related symptoms (e.g., rash from an accelerometer belt or delayed post-walking musculoskeletal soreness) and unrelated events (e.g., previously scheduled knee joint replacement or ACL surgery). Second, individual symptoms were summarized according to categories describing the general nature of the AE (e.g., cardiovascular, musculoskeletal, motor vehicle accident). When appropriate, subcategories were developed to more precisely describe specific symptoms or conditions with a high prevalence (e.g., uncontrolled blood pressure, angina, etc. for CVD adverse events).

Symptoms were then rated according to medical severity using a dichotomous rating of *serious *or *non-serious *clinical severity (Table 1). Serious AE symptoms were defined as symptoms that posed a threat to the health of the participant and which mandated study suspension for immediate medical treatment or consultation with a physician as consistent with most IRB guidelines. Non-serious events were categorized as meaningful events of moderate or minor clinical severity. For the Results section, AE's were analyzed and summarized according to four categories: 1) serious study-related events, 2) non-serious study related events 3) serious non-study related events, and 4) non-serious non study related events.

**Table 1:** Summary of study and non-study related adverse events symptoms

**Severity, n **(%)	**Study Related**	**Non-study Related**
Serious	2 (1.4)	46 (31.5)
Non-Serious	17 (11.6)	81 (55.5)

### Data analysis

Basic descriptive statistics were used to summarize the findings. Means and standard deviations were calculated for age, BMI, and number of presenting health conditions. Frequency counts and percentages were used to describe baseline demographic characteristics and health conditions as well as the type and severity of AE's reported during the course of the study.

## Results

### Participants

Between study inception on August 29, 2005 and November 27, 2006, 274 individuals had been recruited and enrolled in the Veterans Walk for Health trial. Of the enrolled participants, 244 had been randomized to the three study arms. One of the original six sites stopped recruiting due to difficulties implementing study protocols; only data from participants recruited and *enrolled *at the remaining five sites are analyzed here. At baseline, the mean age of participants was 56.3 years (SD = 9.9) with a range between 30 and 84 years. Table 2 provides a brief summary of participant demographic characteristics. The sample reported a high level of education with almost 81% of participants attaining at least some college-level education, and the majority reported an annual income of less than $40,000.

**Table 2:** Demographic characteristics of participants

	**N**	**%**
Ethnicity (N = 272)*, **		
White/Hispanic	182	66.9
African American	74	27.2
Native American or Alaskan Native	14	5.1
Asian	1	0.4
Pacific Hawaiian or other Pacific Islander	1	0.4
Not given	5	1.8
		
Education (N = 272) **		
Less than High School	10	3.7
High School Graduate	43	15.8
Some College	128	47.1
College Graduate	63	23.2
Graduate School	28	10.3
		
Income (N = 272) **		
< $20,000	89	32.7
$20,000 – $39,999	71	26.1
$40,000 – $59,999	46	16.9
$60,000 – $80,000	31	11.4
> $80,000	26	9.6
Not given	11	4.0
		
Smoker **	44	16.9
		
**Mean Body Mass Index (BMI) **per participant^†^	36.1 ± 5.3	

* Total frequency exceeds 100% due to three individuals who endorsed two or more ethnicities.

** Missing data for two individuals reduced enrolled sample n to 272 for this descriptive analysis.

^† ^Analysis based on N = 274.

A total of 387 individuals were physician- or self-referred to the study. Seventeen individuals could not be reached by phone for screening. Of the 370 persons who underwent screening, 49 were excluded for the following reasons: unable to walk a block (n = 13); BMI < 28 (n = 15); were not sedentary (n = 17); female (n = 2); already using a pedometer (n = 1); and mental incapacity (n = 1). A total of 33 individuals were excluded due to lack of interest in becoming physically active or participating in a walking program. Another 14 individuals refused to sign the informed consent form resulting in 274 participants enrolled in the study. Subsequently, 30 participants who were enrolled dropped out before randomization. Overall, 29% of prospective participants were excluded during recruitment with an additional 8% of eligible participants failing to return after enrollment. A total of 244 participants were randomized to the three study arms. Because AE tracking was initiated upon study enrollment, all demographic and AE characteristics summarized in this study describe data for enrolled participants including those individuals who dropped out of the study between enrollment and randomization.

Table 3 summarizes the high prevalence of sample disease morbidity. Participants reported an average of 5.2 health conditions (SD = 2.3) pertaining to a specific chronic disease or functional impairment. Almost 74% of the sample reported four or more health conditions while fewer than 14% cited two or fewer health problems. Baseline body mass index (BMI) assessments averaged 36.1 (SD = 5.2). The effect of the high levels of disease and potentially disabling conditions is reflected by the finding that less than 15% of respondents rated their health as *very good *to *excellent*.

**Table 3:** Participant baseline health characteristics at baseline assessment

	**Frequency**	**%**
Health Status (N = 272)*		
Very Good to Excellent	39	14.2
Good	111	40.5
Poor to Fair	99	40.9
Not given	10	4.4
		
Baseline Comorbidities (N = 272)*		
Hypertension	192	70.6
High Cholesterol	178	65.4
Depression, anxiety, or other mood disorder	159	58.5
Osteoarthritis	142	52.2
Diabetes	121	44.5
Sleep disorders	118	43.4
Chronic pain	117	43.0
Hearing problems	95	34.9
Angina	63	23.2
Lung disease, emphysema, asthma, or bronchitis	58	21.3
Heart Attack	34	12.5
Cataracts	34	12.5
Cancer (non-skin)	24	8.8
Congestive Heart Failure	22	8.1
Stroke	18	6.6
Hip or knee joint replacement surgery	18	6.6
Glaucoma	14	5.1
Osteoporosis	14	5.1
Parkinson's disease	1	0.4
		
**Mean Number of Comorbidities **per participant *	5.2 ± 2.3	

* Missing data for two individuals reduced enrolled sample n to 272 for this descriptive analysis.

### Adverse events

As of November 27, 2006, 121 AE reports were submitted for 87 participants (32% of all enrolled study participants), representing 146 discrete symptoms that are summarized in Tables 4 and 5. Adverse event statistics summarized in this study reflect 1109.8 cumulative months of participant exposure to study-related walking participation. A total of 24 adverse symptoms were filed in 20 reports prior to randomization by 20 enrolled participants. Table 5 highlights that AE's unrelated to study participation constituted 87% (n = 127) of all adverse symptoms. Approximately 53% of all AE reports were generated by 11% of the enrolled study participants (n = 29). Tables 4 and 5 show that CVD symptoms and musculoskeletal conditions represented the most common medical problems experienced by participants over the course of the study.

**Table 4:** Clinical typology of adverse events symptoms

**Type of Medical Event**	**Number of Adverse Events**
**Cardiovascular**	
High/Low blood pressure	32
Angina/Chest tightness	9
Orthopnea, low extremity edema	7
Shortness of breath	9
Light-headedness, dizziness	12
Diaphoresis	2
Stroke, myocardial infarction	2
Other CVD	1
	
	**74**

**Metabolic/Endocrine**	
Sudden weight gain	3
Hypo-, Hyperglycemia	5
Hypo-, Hyperkalemia	2
	
	**10**

**Neuro-Psychological**	
Neuro-motor	4
Mood Disorder	4
	
	**8**

**Musculoskeletal**	
Laceration, scrape, bruises	3
Muscle pulls, strains, sprains, soreness	13
Bone or joint trauma/fracture	11
	
	**27**

**Gastrointestinal**	
Gastric reflux	1
Rectal bleeding	2
	
	**3**

**Infectious Disease**	
Dermatological (fungus, blister, boil)	4
Viral, bacterial, fungal, or insect	11
	
	**15**

**Medical Events**	**137**
	
**Motor Vehicle Accident**	**3**
	
**Surgical Comorbidity**	**6**

**Total Number of discrete symptoms**	**146**

*Note*. These symptoms represent 121 individual adverse event reports from 87 separate individuals. Thus, multiple symptoms could be reported on an individual report and some participants experienced 2 (n = 24) or 3 (n = 5) adverse events during the trial period summarized for this study.

**Table 5:** Classification of adverse events by cause and relatedness to study intervention

	**Study Related**		**Non-study Related**		
		
**Category, Freq: n **(%)	**Serious**	**Non-Serious Minor-Moderate**	**Serious**	**Non-Serious Minor-Moderate**	**Total**
**Cardiovascular**	**2 **(1.4)	**4 **(2.6)	**23 **(15.8)	**45 **(30.8)	**74 **(50.6)
**Musculoskeletal**	**0 **(0.0)	**9 **(6.2)	**3 **(2.1)	**15 **(10.3)	**27 **(18.6)
**Metabolic-Endocrine**	**0 **(0.0)	**0 **(0.0)	**6 **(4.1)	**4 **(2.6)	**10 **(6.7)
**Infectious Disease**	**0 **(0.0)	**3 **(2.1)	**4 **(2.7)	**8 **(5.5)	**15 **(10.3)
**Psycho-Neurological**	**0 **(0.0)	**1 **(0.7)	**4 **(2.7)	**3 **(2.1)	**8 **(5.5)
**Gastrointestinal**	**0 **(0.0)	**0 **(0.0)	**0 **(0.0)	**3 **(2.1)	**3 **(2.1)
**Motor Vehicle Accident**	**0 **(0.0)	**0 **(0.0)	**2 **(1.4)	**1 **(0.7)	**3 **(2.1)
**Surgical**	**0 **(0.0)	**0 **(0.0)	**4 **(2.7)	**2 **(1.4)	**6 **(4.1)

**Total**	**2 **(1.4)	**17 **(11.6)	**46 **(31.5)	**81 **(55.5)	**146 **(100.0)

*Note*. Serious AE's were defined consistent with most IRB definitions as a 1) death; 2) a life threatening experience; 3) hospitalization or prolongation of hospitalization; 4) persistent significant disability or incapacity; or 5) an event that jeopardizes the subject and may require medical or surgical treatment to prevent one of the preceding outcomes. Non-serious AE's consisted of both minor and moderately serious AE's. Moderately serious AE's were defined as concerning new medical symptoms (not imminently life threatening) or acute events that either required suspension until clearance by a physician to rule out potential for Serious AE's (cardiopulmonary dysfunction) and insure optimal patient self-care (e.g., medication, blood pressure, or blood sugar regulation) or, events causing voluntary cessation of walking by the participant until the aggravating event passed (e.g., illness, serious musculoskeletal injury, motor vehicle accident). Minor AE's were defined as events not necessitating study suspension and causing minor discomfort or inconvenience to the participant as a result of study participation (e.g., sore muscles, blisters, equipment malfunction).

Only one serious study-related AE report has been reported to date. This report was submitted by a single individual who experienced the two symptoms of dizziness and shortness of breath following a walk. These symptoms were subsequently attributed to atrial fibrillation that resulted in a brief hospitalization for observation. This individual was suspended for three days before obtaining medical clearance from his physician to resume study participation.

Approximately 12% of total adverse symptoms (n = 17) were non-serious study related AE's with minor musculoskeletal complaints constituting most of these events. Five of the nine musculoskeletal AE's reported related to bone or connective tissue pain in the foot, heel, or knee that occurred after walking. The remaining four musculoskeletal events pertained to post-activity muscle cramping or muscle soreness. Of the four non-serious cardiovascular symptoms, three involved shortness of breath after walking and one concerned light-headedness after a walk. Three non-serious study-related dermatological events were recorded including one participant submitting two separate reports of a rash caused by wearing a study accelerometer belt and another participant reporting walking-induced blisters. Lastly, one minor neurological complaint was the occurrence of low back pain aggravated by a walk. Three participants were suspended for an average of 12.7 days (SD = 3.2) for non-serious study related events; one participant who had experienced a non-serious walking related event withdrew for medical reasons.

Serious AE's not related to the study comprised 31.5% (n = 46) of all the reported AE symptoms documented. Within this category, cardiovascular events represented 50% (n = 23) of reported symptoms with most participants presenting to the emergency room with two or more symptoms per incident. Serious cardiovascular incidents included six reports of angina, four cases of dizziness or light-headedness, and three cases reported for each of the symptoms of orthopnea, shortness of breath, and serious hypertension (i.e., systolic pressure > 160 or diastolic pressure > 95). Additionally there were two reports of both strokes and diaphoresis. One of the participants who had a stroke subsequently died.

The remaining twenty-three serious AE's not related to study participation reflected a wide range of medical events. Metabolic abnormalities were the most common problem including six cases of hypoglycemia, hypokalemia, or sudden weight gain. Four participants were hospitalized for severe depressive symptoms, four participants were hospitalized for bacterial or viral infections, and four participants underwent surgical procedures: one carotid artery occlusion, one liver transplant, one ACL repair for a pre-existing condition, and one knee joint replacement for a pre-existing condition. Three serious musculoskeletal reports included accidental trauma to a finger, motor vehicle accident-related back pain, and electric burns. Finally, two participants reported moderate psychological and musculoskeletal trauma from motor vehicle accidents resulting in temporary suspension. A total of 15 participants were temporarily suspended for serious AE's not related to the study for average suspension duration of 28.3 days (SD = 42.0) before obtaining physician permission to resume walking. Seventeen participants who experienced serious AE's unrelated to walking withdrew from the study.

Non-serious AE's not related to study participation included events of minor to moderate severity that represented 56% of all adverse symptoms. Cardiovascular symptoms made up 45 of the 81 non-serious symptoms in this category. These CVD symptoms can be summarized as: 29 reports of poorly regulated blood pressure, 6 cases of dizziness or light-headedness, 3 complaints of angina, 2 cases of shortness of breath, 2 cases of orthopnea, 2 cases of peripheral edema, and 1 report of abnormal cardiac stress test results.

In addition to non-serious CVD events unrelated to the study, a diverse number of non-cardiac medical events were reported. Fifteen non-serious musculoskeletal injuries unrelated to walking were reported including: five cases of muscle or joint soreness; four minor falls causing bruising of bones or joints; three significant bruises or lacerations; one case of shin splints; one report of a swollen knee joint; and one ankle sprain. Eight cases of infectious agents were reported including a skin boil, influenza, painful reactions to insect stings/bites, and upper respiratory tract infections. Metabolic or endocrine events included three cases of poorly regulated blood sugars and one case of sudden weight gain. Other minor non-study related AE's included three cases of neurological events due to instability caused by vertigo and mild sciatica, two cases of rectal bleeding, one report of gastric reflux, and one motor vehicle accident. Two outpatient surgeries for cataracts and a hernia were also non-serious AE's unrelated to the study. Non-serious AE's unrelated to the study resulted in 31 participants being suspended for an average of 27.3 days (SD = 33.7) before resuming study participation, and 9 participants who experienced non-serious AE's unrelated to walking withdrew from the study.

## Discussion

Our study demonstrates that chronically diseased individuals can safely start an unsupervised home-based walking program with low risk of serious PA-related AE's. Despite a high prevalence of chronic disease morbidity in our sample, only one participant reported a serious case of atrial fibrillation related to walking. Only thirteen percent of AE symptoms were study-related and were predominantly non-serious cardiovascular or musculoskeletal symptoms of minor clinical severity. In contrast, almost 90% of all AE's were health problems unrelated to the walking. The majority of non-study related AE's were non-serious symptoms that were associated with poor management of CVD and diabetes symptoms, such as uncontrolled hypertension or poorly regulated blood glucose levels. However, the prevalence of AE's unrelated to the study that lead to temporary suspensions from walking underscores the fact that chronically diseased older adults face a wide variety of medical events that may act as barriers to exercise. These events are quite diverse and can range from acute illnesses to emergency surgeries and severe depressive episodes.

The impetus for this paper was to contribute to the formation of an evidence base that describes the incidence of AE issues related to community-based, lifestyle activity interventions for high-risk individuals. A recent review on this topic by investigators representing the Behavioral Change Consortium (BCC) of the National Institutes of Health (NIH) provided preliminary descriptive findings with which to compare our results [20]. The BCC review offered similar results to the present study and reported that no serious study-related AE's occurred in response to moderate PA interventions delivered in eleven RCT's involving over 5000 participants. Among three BCC studies providing detailed AE information, the review found that 75% to 87% of AE's were unrelated to study participation and consisted of transient minor or moderate clinical symptoms similar to those reported in Veterans Walk for Health. Despite differences in sample characteristics and intervention protocols, these findings collectively suggest that moderate-intensity PA interventions are safe but morbidity unrelated to exercise participation is a critical challenge to sustained PA participation.

Recently published findings from the Lifestyle Interventions and Independence for Elders pilot trial (LIFE-P) [25] are among the first well-documented AE findings in a high-risk medical population performing lifestyle activity and provide a basis of comparison for findings in the present study. The LIFE-P study was a RCT designed to pilot study protocols for a trial comparing a moderate-intensity PA intervention to a health education program in sedentary adults between the ages of 70 and 89 years. LIFE-P protocols required close monitoring of AE's for participants in both the PA intervention and sedentary control groups. Like Veterans Walk for Health, there was a high prevalence of disease morbidity among the sample of 424 participants; however, the average age of LIFE-P participants was 20 years greater than our sample. LIFE-P results showed no significant differences in the occurrence of AE's between the PA intervention group and the health education control group except for a higher prevalence of abnormal heart rhythm reports in the active group. In the younger Veteran's Walk for Health sample, only one serious study-related event occurred and, like LIFE-P, this event was related to an abnormal heart rhythm. Collectively, results from both the LIFE-P and Veteran's Walk for Health studies suggest that a moderate-intensity lifestyle PA like walking can be safely performed without supervision and that medical adverse events are a common occurrence that must be monitored to ensure at-risk individuals derive the benefits of *sustained *PA participation.

Veterans Walk for Health reflects a pragmatic approach to preventive medicine that views exercise therapy as a fundamental component of disease management in primary care settings. Recent reviews have documented the prospective and RCT evidence in support of exercise as a cost-effective, adjunct therapy for both the prevention and management of morbidity and mortality associated with several chronic diseases, including obesity [11,13,26-31]. The greatest benefit in mortality reduction is evidenced by those individuals who transition from a sedentary to moderately active lifestyle, even for those with high BMI or those who initiate exercise late in life [2,26,32,33]. However, there is growing recognition that public health efforts to promote increased levels of PA through lifestyle activities are paradoxically undermined by barriers created by national exercise guidelines.

A growing number of medical professionals and PA researchers have identified the extensive pre-activity screening process recommended by exercise guidelines as a significant barrier to exercise initiation in older and at-risk adults. Exercise guidelines disseminated by medical organizations like the American Heart Association (AHA) [21] and the American College of Sports Medicine (ACSM) [34,35] were designed to identify health conditions that are contra-indications to vigorous PA in order to minimize the risk for severe cardiovascular events in response to vigorous intensity exercise. The practical utility of these pre-activity screening procedures has been questioned as a number of studies have revealed that the risk of such events is quite low while information obtained is poor in predicting future severe cardiovascular AE's in older and chronically ill individuals [1,6,36-38]. Some critics have observed that the economic and logistical costs associated with conducting these procedures creates an unnecessary burden on both patients and the medical system while undermining individuals' exercise motivation by implying that PA is hazardous [15,16,20]. Given the number of sedentary adults screened out by this approach, [16] the costs of maintaining this approach far exceed the risk-protective benefit gained from screening at a population level [6,15,16,20,38-40]. Rigorous pre-activity screening also appears unnecessary for those who desire to start a lifestyle activity program consisting of mild- to moderate-intensity activities like walking. The risk for serious cardiovascular-related AE's occurring as a result of walking is considered similar to performing normal activities of daily living, [1] yet current screening guidelines do not provide a practical framework to help older and chronically diseased individuals overcome the ubiquitous medical problems unrelated to exercise that can undermine activity adherence. Recognizing the weaknesses in early guidelines, two expert medical panels have stated that exercise testing is unnecessary for adults that are asymptomatic for CVD and want to initiate a moderate PA program [39,41] with the caveat that these individuals collaborate with their doctors about their exercise program.

Veterans Walk for Health employed an AE monitoring system that encouraged participants to work with VA physicians to manage medical problems identified during study participation and to grant medical authorization to resume study participation when doctors deemed it safe for an individual to resume walking. The VA medical system offers a unique context in which to test innovative interventions that have broader implications for the American public. Development of healthy lifestyle programming is a priority in the VA medical system where the prevalence of chronic disease morbidity and functional disability among veterans is currently above national averages [42,43]. Continuous improvement of patient care and safety are core missions of the VA, and treatment of obesity and obesity-related disorders became a high priority in 2004. Veterans Walk for Health reflects this initiative to develop and evaluate safe and sustainable lifestyle interventions to improve health-related quality of life among veterans.

The screening and safety approach utilized in Veterans Walk for Health offers one model with several unique strengths for promoting PA in a primary care setting. Reflecting a paradigm shift in exercise prescription from the snapshot approach using stress testing to predict activity participation risk, the current study utilized an ongoing monitoring and assessment process between the participant and the intervention team with backup from the participant's physician. We feel the medical clearance forms [see Additional files 1 and 2] provide a pragmatic means to recruit high-risk populations that do not impose undue burden on either participants or their physicians yet give physicians alternatives to assess the cost-benefit ratio of participant PA participation. The AE checklist [see Additional file 3] was significantly enhanced during the first weeks of the trial to improve the accuracy and reliability of AE reporting among sites. To encourage consistency in AE reporting, the form was streamlined to explicitly differentiate and categorize AE's that merit study suspension and require medical clearance versus events where consultation with a primary care provider is advocated or no suspension is needed. Moreover, mandatory suspensions (as well as voluntary suspensions by the participant) are clearly highlighted in a special box to help site coordinators identify and track the medical status of suspended participants. Study personnel were also required to ask participants whether AE's occurred as a result of performing the walking intervention.

A strength of the present study was the utilization of the centralized patient record systems of the VA medical system to link physicians, dietitians, and behavioral researchers. This centralized medical record system allowed us to closely track adverse medical events that occur to participants during the study. Close tracking of adverse medical events also allowed us to get suspended participants back in the walking program quickly after medical issues were resolved. Second, rather than recruiting asymptomatic or healthy participants, the study deliberately recruited high-risk individuals from regions across the U.S. to enhance external validity and to test the effectiveness of this approach in various primary care settings across the U.S. Third, extensive pre-activity screening was viewed as redundant since these participants were already under the care of a physician aware of salient health risks. Our inclusion criteria required that participants be under physician care during intervention participation so that the physician would not only provide one-time medical clearance at baseline, but also be available for helping the participant overcome transient medical problems that might affect compliance with the six-month intervention. As our present findings show, older and chronically ill adults commonly experience medical events of varying severity that may be barriers to PA participation. Our study supports previous assertions that primary care physicians can play a vital role in promoting safe exercise participation in patients performing unsupervised activity [44].

Our findings must be viewed within the limitations of the study design. The absence of a non-active control group limits the comparison between AE's that occurred in a comparable group of sedentary individuals versus the walkers in Veterans Walk for Health. Also, while primary care practices may be an ideal setting to help adults at risk for chronic disease initiate a lifestyle PA program, many providers lack the integrated reporting system developed by the VA and used in the current study to monitor patients. Many hospital systems lack the electronic and centralized patient record system, which permits medical personnel to review a patient's comprehensive medical record to modify an exercise prescription in light of recent changes in medications, fitness, or disability. Although the present study appears to provide support for the premise that lifestyle PA interventions can be safely implemented among older and at-risk adults, our findings also underline the need for primary care access to overcome the inevitable medical events associated with an aging and increasingly high-risk population.

## Conclusion

Little is known about the risk for adverse events among high-risk individuals in response to unsupervised lifestyle activity programs that emphasize moderate intensity PA like walking. This study addressed this gap in the literature by targeting a population that, despite high risk for chronic disease morbidity, could benefit from a simple PA intervention. We found that individuals at high risk for CVD could safely start an unsupervised home-based walking program with a low risk for adverse reactions to walking. However, we documented that 87% of our reported adverse events were due to medical events unrelated to walking but likely to occur among an older, diseased population. Helping individuals resume PA after a medical problem is challenging, but we believe it should be a priority. Recognizing that exercise interventionists can interface with primary care professionals to help chronically diseased individuals overcome these medical barriers,[15,20,44] we described our safety protocols as one approach to address this unmet health service need. There is increasing appreciation that safety screening of PA program participation is an ongoing process [20]. An essential and overlooked role of this process is not only to document medical problems associated with PA participation, but also to facilitate solutions that help individuals cope with the health barriers to PA program adherence and thus, avoid the deleterious effects of inactivity. Further research is needed to assess the risk of adverse events in community-based PA interventions in high-risk populations and to document methods of helping individuals overcome transient health problems to resume the level of PA necessary to confer health protective benefits.

## Competing interests

The author(s) declare that they have no competing interests.

## Authors' contributions

DEG made significant contributions to the design, analysis and interpretation of data, and drafting and revising the manuscript for intellectual content. ARL and RGH assisted in the acquisition, management, and analysis of data. JCL was instrumental in contributing to the original design and conception of the trial and for obtaining funding. CRR made significant intellectual contributions to the conception and design, interpretation of data, and drafting and revision of the manuscript as well as obtaining funding support as primary investigator.

## Supplementary Material

Additional File 1 Medical clearance form. This is a copy of the medical clearance form that was used in the Veterans Walk For Health Study that participant's health care provider signed permitting them to join the study. Click here for file

Additional File 2 Medical clearance form after AE. This is a copy of the medical clearance form that was used in the Veterans Walk for Health Study that enabled a participant to resume a walking program after experiencing an adverse event. Click here for file

Additional File 3 Adverse event checklist. This is a copy of the adverse event checklist that was used in the Veterans Walk for Health Study to record a participant's adverse event. Click here for file
