# Supplementary material for: Adverse events among high-risk participants in a home-based walking study: a descriptive study
Source: Int J Behav Nutr Phys Act. 2007 May 23;4:20. doi: 10.1186/1479-5868-4-20 (PMC1891313; doi:10.1186/1479-5868-4-20)
Supplement: Additional File 1 — Medical clearance form. This is a copy of the medical clearance form that was used in the Veterans Walk For Health Study that participant's health care provider signed permitting them to join the study. [file 1479-5868-4-20-S1.doc]

### Additional file 1. Medical clearance form

VETERANS WALK FOR HEALTH STUDY

MEDICAL CLEARANCE FORM

**_______________________________________________________________________**

I give the research staff from the Veterans Walk for Health Study permission to contact my doctor, Dr. ______________________________ to obtain medical clearance for me to participate in the Veterans Walk for Health Study.

Participant’s Signature _________________________________ Date:_____________

**________________________________________________________________________**

Date: _______________

To: Dr. _______________________

We are requesting medical clearance for your patient _____________________________ to participate in the Veterans Walk for Health study, a randomized controlled trial of a walking intervention for people with cardiovascular disease risk factors.

For most individuals with cardiovascular disease risk factors, starting a walking program significantly decreases the risk of premature death and adverse cardiac events. However, for some individuals, the risks associated with starting a walking program may outweigh the benefits. Examples of such very high-risk patients include a patient with unstable angina who is currently undergoing an evaluation for possible revascularization or a patient with decompensated congestive heart failure.

For the above named patient, please check the appropriate line below and sign. Your patient will not be able to start the walking program until this form is completed.

____ This patient is an appropriate candidate to start a walking program

____ This patient is currently at too high risk to start a walking program

Details ______________________________________________

____ I need to schedule an appointment or conduct further evaluation before giving

medical clearance for this patient to start a walking program.

____ I do not know this patient and cannot evaluate.

Physician Signature ___________________________________ Date _____________
