# Supplementary material for: Adverse events among high-risk participants in a home-based walking study: a descriptive study
Source: Int J Behav Nutr Phys Act. 2007 May 23;4:20. doi: 10.1186/1479-5868-4-20 (PMC1891313; doi:10.1186/1479-5868-4-20)
Supplement: Additional File 2 — Medical clearance form after AE. This is a copy of the medical clearance form that was used in the Veterans Walk for Health Study that enabled a participant to resume a walking program after experiencing an adverse event. [file 1479-5868-4-20-S2.doc]

### Additional file 2. Medical clearance form after AE

VETERANS WALK FOR HEALTH STUDY

MEDICAL CLEARANCE FORM

FOR RESUMING A WALKING PROGRAM

**_______________________________________________________________________**

I give the research staff from the Veterans Walk for Health Study permission to contact my doctor, Dr. ______________________________, to obtain medical clearance for me to resume a walking program as part of the Veterans Walk for Health Study.

Participant’s Signature _________________________________ Date:_____________

**________________________________________________________________________**

Date: _______________

To: Dr. _______________________

We are requesting medical clearance for your patient _____________________________ to resume a walking program as part of the Veterans Walk for Health Study.

His walking program has been discontinued because of the following adverse event:

For the above named patient, please check the appropriate line below and sign. Your patient will not be able to resume the walking program until this form is completed.

____ This patient is an appropriate candidate resume the walking program.

____ This patient is currently at too high risk to resume the walking program.

Details ______________________________________________

____ I need to schedule an appointment or conduct further evaluation before giving

medical clearance for this patient to resume the walking program.

Physician Signature ___________________________________ Date _____________
