# Supplementary material for: Adverse events among high-risk participants in a home-based walking study: a descriptive study
Source: Int J Behav Nutr Phys Act. 2007 May 23;4:20. doi: 10.1186/1479-5868-4-20 (PMC1891313; doi:10.1186/1479-5868-4-20)
Supplement: Additional File 3 — Adverse event checklist. This is a copy of the adverse event checklist that was used in the Veterans Walk for Health Study to record a participant's adverse event. [file 1479-5868-4-20-S3.doc]

**Additional file 3. Adverse event checklist**

Veterans Walk for Health

Adverse Events Checklist

Participant Enrollment ID: ____ ____ ____ ____ ____

| Cardiovascular Events | Heart attack Arrhythmia  Angina Stroke /TIA  Syncope other _______________________________ | Requires suspension until MD clearance. Inform site PI and Ann Arbor coordinating center. |
| --- | --- | --- |
| New Concerning Symptoms | *either*  Short of breath Light headed/dizzy Systolic BP > 160 at visit  Diaphoresis Orthopnea *or*  Chest pain Lower ext. edema Diastolic BP > 95 at visit  Other___________________________________________________ | Requires suspension until MD clearance. Inform site PI and Ann Arbor coordinating center. |
| Other Significant Adverse Events | Motor Vehicle Accident Serious Musculoskeletal Injury  Bacterial Infection on Antibiotics Problems with medication  Problems with low or high blood sugar Dehydration  Other ___________________________________________________ | Contact with PCP recommended. Participant can resume walking when feels ready. |
| Minor Adverse Events | Minor Musculoskeletal injury Problems from pedometer  Lacerations, scrapes or blisters Problems from accelerometer  Sore muscles  Other ___________________________________________________ | No suspension required. |

Describe the adverse event in detail: Event Date: __________________________

___________________________________________________________________________

___________________________________________________________________________

___________________________________________________________________________

Is the Event Serious? YES NO

(Death; a life threatening experience; hospitalization or prolongation of hospitalization; persistent of significant disability or incapacity; congenital anomaly and/or birth defects; or an event that jeopardizes the subject and may require medical or surgical treatment to prevent one of the preceding outcomes.)

Is the Event Unexpected? YES NO

(Any adverse event and/or reaction, the specificity or severity of which is not consistent with the informed consent. Further, it is not consistent with the risk information described in the general investigational plan or proposal.)

Is the Event Related? NO UNLIKELY POSSIBLY PROBABLY YES

[The result of: (a) the interventions and interactions used in the research; (b) the collection of identifiable private information in the research.]

Was the participant on a walk when the event occurred? YES NO

If event was cardiac (ex. chest pain, shortness of breath) did it occur within 6 hours after a walk? YES NO

Has the participant been seen by a physician for this problem?_______________________________________

Site PI Informed? YES NO

Participant suspended? YES NO

If yes, date suspended:___________

Med. Clr. to resume? YES NO

If yes, date resumed:___________

Ann Arbor informed? YES NO (Who?_____________________)

Staff initials ____________ Date __________ Time ___________
